# Global diversity in the *TAS2R38* bitter taste receptor: revisiting a classic evolutionary PROPosal

**DOI:** 10.1038/srep25506

**Published:** 2016-05-03

**Authors:** Davide S. Risso, Massimo Mezzavilla, Luca Pagani, Antonietta Robino, Gabriella Morini, Sergio Tofanelli, Maura Carrai, Daniele Campa, Roberto Barale, Fabio Caradonna, Paolo Gasparini, Donata Luiselli, Stephen Wooding, Dennis Drayna

**Affiliations:** 1 National Institute on Deafness and Other Communication Disorders, NIH, Bethesda, MD 20892, USA; 2 Department of Biological, Geological and Environmental Sciences BiGeA, Laboratory of Molecular Anthropology and Centre for Genome Biology, University of Bologna, via Selmi 3, 40126 Bologna, Italy; 3 Institute for Maternal and Child Health, IRCCS “Burlo Garofolo”, University of Trieste, 34137 Trieste, Italy; 4 Division of Experimental Genetics, Sidra Medical and Research Center, Doha, Qatar; 5 Division of Biological Anthropology, University of Cambridge, CB2 1QH, Cambridge, UK; 6 University of Gastronomic Sciences, Piazza Vittorio Emanuele 9, Bra, Pollenzo 12042, CN, Italy; 7 Department of Biology, University of Pisa, Via Ghini 13, 56126 Pisa, Italy; 8 Biological, Chemical and Pharmaceutical Sciences and Technologies Department, STEBICEF, Università degli Studi di Palermo, V.le delle Scienze, Edificio 16, 90128 Palermo, Italy; 9 Health Sciences Research Institute, University of California at Merced, 5200 North Lake Road, Merced, CA 95343, USA

## Abstract

The ability to taste phenylthiocarbamide (PTC) and 6-n-propylthiouracil (PROP) is a polymorphic trait mediated by the *TAS2R38* bitter taste receptor gene. It has long been hypothesized that global genetic diversity at this locus evolved under pervasive pressures from balancing natural selection. However, recent high-resolution population genetic studies of *TAS2Rs* suggest that demographic events have played a critical role in the evolution of these genes. We here utilized the largest *TAS2R38* database yet analyzed, consisting of 5,589 individuals from 105 populations, to examine natural selection, haplotype frequencies and linkage disequilibrium to estimate the effects of both selection and demography on contemporary patterns of variation at this locus. We found signs of an ancient balancing selection acting on this gene but no post Out-Of-Africa departures from neutrality, implying that the current observed patterns of variation can be predominantly explained by demographic, rather than selective events. In addition, we found signatures of ancient selective forces acting on different African *TAS2R38* haplotypes. Collectively our results provide evidence for a relaxation of recent selective forces acting on this gene and a revised hypothesis for the origins of the present-day worldwide distribution of *TAS2R38* haplotypes.

More than 70 years ago A.L. Fox reported that phenylthiocarbamide (PTC) tastes extremely bitter to some people (defined as “tasters”) but not bitter at all to others (“non-tasters”)^1^,^2^. Since then, numerous family, twin and population studies have shown that the inability to taste PTC is inherited in a nearly Mendelian recessive manner^3^,^4^,^5^. In more recent studies, the use of the chemically similar 6-n-propylthiouracil (PROP) has often been substituted for PTC because of its ability to generate similar taste responses in humans and its better known toxicity profile^6^. In 2003, a locus that explained approximately 75% of the variation in PTC sensitivity was identified on chromosome 7^4^. At this locus, variation in the *TAS2R38* bitter receptor gene was subsequently found to underlie all of the bimodal distribution of this phenotype and to explain >70% of the total phenotypic variance^7^. *TAS2R38* is a member of the TAS2R bitter taste receptor gene family, which in humans consists of 25 functional genes and 11 pseudogenes, many of which show signatures of natural selection^8^,^9^,^10^,^11^. Three single nucleotide polymorphisms (*rs714598*, *rs1726866*, *rs10246939*) at positions encoding amino acids 49, 262 and 296 represent the most common variant alleles of *TAS2R38*, and comprise the “taster” PAV (Proline, Alanine, Valine) and “non-taster” AVI (Alanine, Valine, Isoleucine) haplotypes. In addition, two rare (frequency <5%) (AAV and AAI) and two extremely rare (frequency <1%) (PAI and PVI) haplotypes have been identified. The other two possible haplotypes (AVV and PVV) have been individually reported in two studies^12^,^13^ but not otherwise observed.

A longstanding question has been the reason for the presence of two high-frequency haplotypes in worldwide populations. Because the perception of bitter taste is thought to protect us from the ingestion of toxic substances, how could the presumably non-functional AVI haplotype come to high frequency in populations worldwide? Analyses of the frequency distribution of *TAS2R38* haplotypes in different populations showed significant positive values for Tajima’s D statistic, low F_ST_ values (0.001–0.05) and a deep coalescent time (TMRCA ~1 million years old) for this locus^9^,^14^, providing evidence that balancing selection maintained both the taster and non-taster alleles at high frequency. However, a study of inter-specific variations of bitter taste receptor genes showed that TAS2R genes, and in particular *TAS2R38*, have undergone relaxation of selection in humans when compared with many other mammals^15^.

Balancing selection hypotheses have suggested the possibility that the AVI non-taster allele encodes a fully functional receptor for another hypothetical bitter substance^8^. It has also been suggested that pathogens may have been the real targets of natural selection, since bitter receptors are expressed in the respiratory and enteric system^16^,^17^ and one study suggested that common polymorphisms in the *TAS2R38* gene were linked to significant differences in the ability of the upper respiratory cells to clear and kill bacteria^18^. In addition, a recent study showed that *TAS2R38* genotypes regulate innate immune responses to oral bacteria^19^.

Here we studied the distribution of *TAS2R38* haplotypes in a large number of human populations and available archaic hominid genomes to provide a fine-scale view of worldwide *TAS2R38* diversity, and we applied selection tests to evaluate the processes underlying the evolution of *TAS2R38* haplotypes.

## Results

### Population Genetics of *TAS2R38*

The analysis of the *TAS2R38* gene and surrounding regions (500 bp 5′ + 500 bp 3′) in the 1000 Genomes database showed that 96.07% of the variation in this locus was due to differences within populations, with little existing among continental sub-groups (4.1%) or populations (0.83%). However, geographically diverse populations showed differences at the nucleotide level, and African populations, in particular, carried more polymorphic sites when compared to populations of European, Asian and American ancestry. In addition, both gene and nucleotide diversity were higher in African populations (Supplementary Table S1). This is confirmed by the Wright’s fixation indices calculated between all the analyzed populations, divided in continental groups (F_SC_ = 0.01, F_CT_ = 0.04 and F_ST_ = 0.05; all tests with P < 0.001), indicating a moderate to low level of differentiation at this locus between populations. The three *TAS2R38* SNPs (*rs714598*, *rs1726866* and *rs10246939*) showed approximately the same global F_ST_ values (0.05, 0.06 and 0.04 with P = 0.01, 0.01 and 0.002 respectively). However, when comparing these values to those obtained for SNPs of similar minor allele frequency across the genome, *TAS2R38* F_ST_ values were not outliers (Supplementary Figure S1), and the probability of observing a SNP with F_ST_ < 0.05 in this distribution is 0.34. Taking into account the presence of three SNPs in the same gene with high LD levels (average R^2^ of 0.87), the probability of drawing three linked SNPs showing F_ST_ < 0.05 is 0.29 (0.34*0.87), not statistically lower than that of SNPs in the 5^th^ percentile genome-wide.

We then calculated the global frequency of the *TAS2R38* haplotypes in three population datasets for which we had sequence data for the three common *TAS2R38* variants: 1000 Genomes (2N = 2722), our Italian populations (2N = 2878) and AGVP chromosomes (2N = 456) for which whole-genome sequences were available (2N_tot_ = 6,056). In the combined dataset, the PAV and AVI haplotypes were predominant (50.76% and 42.70%, respectively), followed by AAI (3.39%) and AAV (2.48%). Other haplotypes occurred at very low frequencies, AVV (0.32%), PAI (0.18%), PVV (0.10%) and PVI (0.07%) (Table 1). All the identified haplotypes were in Hardy-Weinberg equilibrium in each analyzed population (P > 0.05, data not shown).

**Figure 1:**
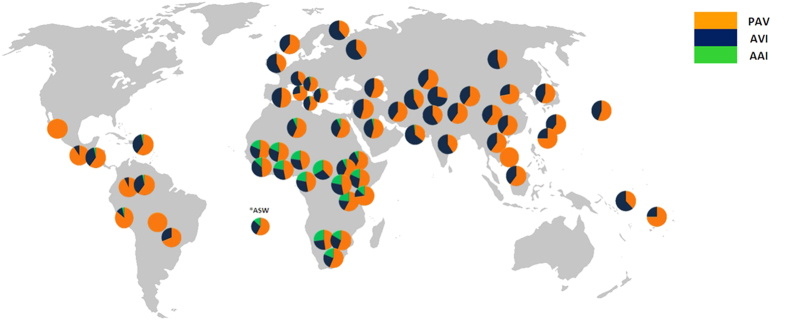
Worldwide distribution of *TAS2R38* PAV, AVI and AAI haplotypes in the studied populations. This map has been modified from its original version (https://commons.wikimedia.org/wiki/File:BlankMap-World-noborders.png).

The worldwide distribution of the PAV, AVI and AAI haplotypes calculated in our entire database (2N_tot_ = 11,178) is shown in Fig. 1. Other than a few exceptions with low frequencies in Central-South Italy (≤1%) and in the Near East (≤5%), the AAI haplotype is uniquely present in Africa, where it occurs at moderate-high frequencies (7–33%). It should be noted that, given the lack of data for *rs714598* in both the Silk Road and the HGDP datasets and of *rs10246939* in some individuals of the AGVP, the percentage of AAI could be slightly overestimated in these populations compared to that of AAV. However, the frequency of AAV in our African and Asian populations proved to be extremely low (0.61% and 0% respectively, Table 1), indicating that any overestimation effect is likely negligible.

Supplementary Figure S2 shows details of the distribution of AAI diplotypes in Africa. The PAV/AAI combination is the most common (ranging from 25 to 100%), with AAI homozygotes being present exclusively in the sub-tropical parts of Africa, where they displayed frequencies of 3–17%. In addition to AAI, the two major haplotypes (i.e. PAV and AVI) showed a different degree of distribution among continents, with AVI being less prevalent in both the African and American continental populations (Chi-square test, P < 0.001).

### Haplotype and Linkage Disequilibrium Analysis

**Figure 2:**
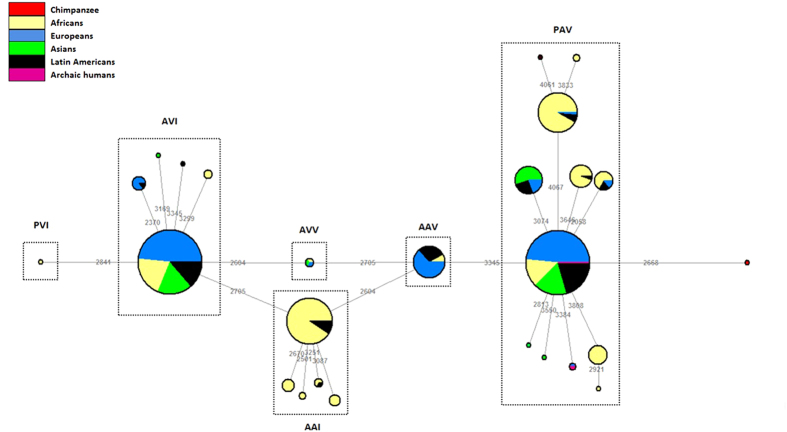
Neighbor-joining haplotype network illustrating the genealogical relationships between *TAS2R38* haplotypes in archaic hominids and modern African, European, Asian and Latin American populations. The position of the chimpanzee (outgroup) is highlighted in red.

The distribution of *TAS2R38* haplotypes, based on nucleotide data from the 1000 Genomes dataset, is represented in the median-joining network shown in Fig. 2. Six different clusters could be observed, with the PAV clade containing the highest number of different sub-haplotypes (N = 11). Our data indicate that the extremely rare (frequency <1%) AVV haplotype occupies an intermediate position between the AVI and the rare (frequency <5%) AAV haplotype. We also find that the archaic hominins (i.e. Neanderthal and Denisovan), lie in the PAV clade, suggesting an ancestral state of this haplotype.

Linkage Disequilibrium (LD) analyses performed on the *TAS2R38* gene and surrounding regions in the 1000 Genomes dataset, confirmed that *TAS2R38 rs714598*, *rs1726866* and *rs10246939* variants are in strong LD (R^2^ = 0.80−0.93, D′ = 0.997−0.998). In addition, a fourth SNP (*rs4726481*) situated in a nearby gene (*MGAM*, maltase-glucoamylase) was found to lie in the same haplotype block and in moderately strong LD (R^2^ = 0.63−0.81, D′ = 0.992−0.994) with the *TAS2R38* SNPs in all populations analyzed. In addition, Asian populations showed substantial LD values (R^2^ = 0.66−0.67, D′ = 0.812−0.833) with another SNP (*rs17162635*) in the same gene. Both the *MGAM* SNPs are located in intronic regions and display different distributions across continents, with rs4726481 having a derived allele frequency (DAF) approaching 0.50 in Africa, Europe, Asia, Latin America and *rs17162635* showing a DAF much higher in Asia (0.29) compared to Europe (0.13), Latin America (0.10) and Africa (0.04).

### Testing neutrality and selection

**Figure 3:**
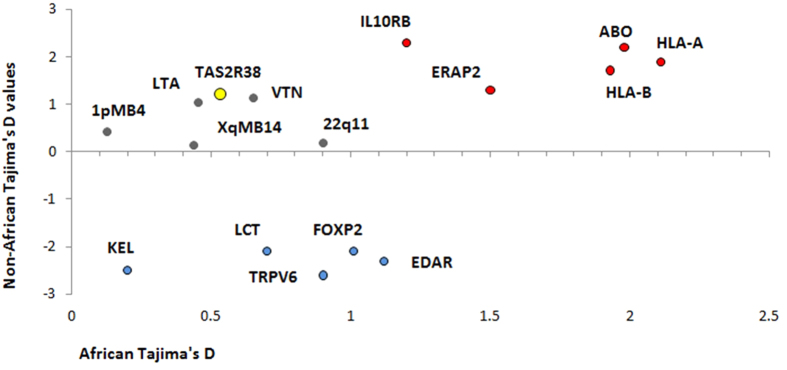
Comparison between Tajima’s D values calculated in African and non-Africans for genes under different selective pressures. Genes under positive selection are represented with blue circles, genes under balancing selection with red circles and neutral regions with grey circles. The position of *TAS2R38* is highlighted in yellow.

We performed several tests to measure the deviation of the genetic differences at the *TAS2R38* locus from neutral expectations using the 1000 Genomes dataset. First, we computed Tajima’s D statistics in different worldwide populations. The coding region of *TAS2R38* gene showed positive, although not significant (P > 0.05) values in all populations examined. Both the flanking 5′ and 3′ regions showed negative and not significant (P > 0.05) values. Moreover, when compared to Tajima’s D values obtained across the genome for coding loci of similar size (1,143 +/− 500 bp) the *TAS2R38* values resided between the 5th and 95th percentiles (Supplementary Figure S3). Expanding the analyzed region to 10,000 bases around these selected loci showed similar results (data not shown). In addition, when compared to Tajima’s D values calculated for genes for which a clear adaptive significance has been suggested, *TAS2R38* clustered with genes considered to be evolutionarily neutral (Fig. 3).

We further explored this using Li’s MFDM, which has been shown to be more robust in the presence of confounding effects such as population size fluctuations and other demographic events. This analysis failed to detect any signs of deviation from neutral expectations in *TAS2R38* (P = 0.63). The same results were obtained when using the HKA test (P = 0.35). We also performed a Bayesian analysis in an effort to detect evidence of balancing selection. The calculated alpha values for *TAS2R38* detected no evidence of selection at *TAS2R38* (−0.5<Alpha<0).

Finally, Supplementary Table S2 shows the window containing *TAS2R38* did not differ from the average heterozygosity levels and did not fall below the 5th or above the 95th percentile

### Simulations of haplotype evolution

We simulated the evolution of the two common *TAS2R38* haplotypes (PAV and AVI), the less common AAI, the rare (frequency <5%) AAV and the rarest (frequency <1%) AVV and PVI haplotypes, under several conditions (see Materials and Methods and Supplementary Information for more details). The only scenario that produced simulated haplotype frequencies similar to current frequencies involved an ancient (e.g. before Out of Africa) balancing selection (s = 0.001) acting on PAV/AVI individuals (Supplementary Figure S4). Under this scenario, the distribution of simulated global haplotype frequencies for the PAV, AVI, AAV, AVV and PVI (0.50 +/− 0.13, 0.47 +/− 0.14, 0.01 +/− 0.05, 0.003 +/− 0.005 and 0.0001 +/− 0.005 respectively) did not differ significantly (P = 0.39) from the distribution of the observed frequencies (0.51 +/− 0.13, 0.43 +/− 0.14, 0.02 +/− 0.07, 0.003 +/− 0.006 and 0.0001 +/− 0.0005 respectively), regardless of the degree of population expansion. However, the simulated frequencies obtained for AAI in African individuals did not match the empirical data (0.02 +/− 0.04 vs 0.13 +/− 0.05, P = 0.001), although the distribution of this haplotype outside Africa was consistent with the frequencies (P = 0.42) obtained from this model (0.02 +/− 0.03 vs 0.03 +/− 0.01). We therefore performed simulations involving different selecting forces acting on AAI in African individuals, both before and after the Out Of Africa (OOA) event. The only scenario that produced results similar to the observed AAI distribution in Africa (0.13 +/− 0.11 vs 0.13 +/− 0.05, P = 0.11) involved a weak (s = 0.001) directional selection force acting on this locus before the OOA event (Supplementary Figure S5).

## Discussion

Although the presence of PTC or PROP has not been documented in nature, chemically similar compounds, known as glucosinolates, are found in common bitter foods, such as brussels sprouts, cabbage and broccoli^20^. For this reason, it has been suggested that a correlation may exist between PROP taster status and dietary intake^21^, which could have important evolutionary consequences, a hypothesis supported by the results of a number of previous studies^22^,^23^,^24^. Other studies have identified relationships between the intensity of PTC/PROP bitterness and the perception of several natural compounds^25^,^26^,^27^,^28^. These correlations generally support the hypothesis that natural selection has been acting on *TAS2R38*, maintaining the AVI haplotype at roughly the same worldwide frequency as the PAV haplotype. In addition to this hypothesis, it has been suggested that the non-taster AVI haplotype may be a functional receptor for another bitter compound^8^ and that pathogens may have been the real targets of natural selection^17^,^18^.

Using the largest sample available, we have confirmed the global predominance of the PAV and AVI haplotypes of *TAS2R38*. We also found that the AVI form is less common in Africa and that the AAI haplotype is primarily present in this continent, as previously reported^9^. The three common *TAS2R38* SNPs (i.e. *rs714598*, *rs1726866* and *rs10246939*) showed low F_ST_ values, indicating little genetic differentiation at these sites. This was previously interpreted^9^ as a footprint of balancing selection that maintained similar frequencies for the alternative alleles at these sites. However in contrast to previous studies^8^,^9^, we failed to detect any recent departures from neutral expectations for the variation at the *TAS2R38* locus and surrounding regions. Although we found positive Tajima’s D values in the *TAS2R38* coding region, these did not reach significance and where similar to those observed at evolutionary neutral loci. When correcting for population stratification and taking into account demographic events with Li’s MFDM test, *TAS2R38* did not show significant P-values, suggesting that these variables may contribute to the observed positive Tajima’s D values. Another study, although based on a much smaller number of individuals (N = 22), previously pointed out this possibility^15^. Other approaches that have previously successfully detected signs of balancing natural selection, including the HKA and Bayescan tests, failed to find any departures from neutrality in our data set. We also note that two other studies applying new methods to detect signatures of balancing selection failed to identify such signatures in *TAS2R38*^29^,^30^.

Here we propose a modified hypothesis for the evolution of *TAS2R38* haplotypes in humans. We suggest ancient balancing selection acting during the early stages of hominin evolution, before the Out-Of-Africa event, that maintained both PAV and AVI alleles at roughly the same frequency. We speculate that both haplotypes were in fact important for detecting potentially toxic bitter compounds found uniquely in the African continent.

Since *TAS2R38* shows no signs of recent departure from neutral expectations, we hypothesize that the modern frequency distribution of the non-taster AVI allele in non-Africans is largely due to recent demographic and population stratification events. This is also supported by our simulations, which confirmed that the existing high frequencies of the PAV and AVI haplotypes outside Africa could have arisen by a series of bottlenecks and population expansion with a relaxation of the selective forces acting on this gene. It has been shown that bottleneck episodes after the out-of-Africa event have strongly contributed to a modification of the genetic structure and of the selective sweeps acting on different human populations^31^,^32^.

The AAI haplotype seems to have a more complex evolutionary history. Its distribution is unique in Africa, aside from a few exceptions in Latin America presumably due to the African admixture in these populations^33^ and in the Near East and southern Italy, regions with strong flow from Africa in historical times^34^,^35^. From our simulations we hypothesize that the AAI haplotype has undergone weak but directional selection, in addition to the balancing selection that previously acted on PAV/AVI. This view is strengthened by a recent work studying a large collection of African populations, where the authors found that African *TAS2R38* haplotypes evolved under a more complex scenario that includes a combination of balancing and directional selective pressures^9^.

Finally, the high LD values between *TAS2R38* and *MGAM* variants suggest that this gene may also have had an effect on the current distribution of the PAV and AVI haplotypes. This gene is involved in pathways and interacts with genes involved in starch and sucrose metabolism, carbohydrate digestion and absorption and galactose and lactose, suggesting a potentially important role for human nutrition.

## Materials and Methods

### Sources of samples and genetic data

Archaic hominids studied in this project consisted of the Altai Neanderthal individual^36^ and the Denisovan hominin from the same site^37^. The modern human subjects consisted of both published databases and newly recruited samples. For the former, we included 1,361 individuals belonging to 17 worldwide populations from the 1000 Genomes Project Phase 3^38^, 942 individuals from 53 different populations from the Human Genome Diversity Project (HGDP)^39^ and 1,428 African individuals from 15 populations of the African Genome Variation Project (AGVP)^40^,^41^. In addition, 419 individuals belonging to 20 communities living along the Silk Road in Caucasus and Central Asia^42^,^43^ and 1,439 adult healthy subjects from 9 different regions within Italy (this study) were included in the analysis. Individuals from different databases but belonging to the same populations were pooled together. A total of 11,178 chromosomes from 105 different worldwide populations were analyzed (Supplementary Table S3). All samples were anonymized, coded identifiers were assigned to them and each donor was given an information sheet. All experimental protocols were approved by the ethical committee of IRCCS-Burlo Garofolo Hospital, by the bioethics committee of Pisa University and by the National Institutes of Health (NIDCD protocol 01-DC-0230) combined Neurosciences/Blue Panel Institutional Review Board. Written informed consent was obtained from all subjects included in the study. All experiments and methods were performed in accordance with the approved guidelines and regulations.

### DNA collection, extraction and genotyping

Samples of saliva from subjects of the Silk Road populations were collected using the Oragene kits and extracted according to the manufacturer’s protocol (Genotek Inc., Kanata, Ontario, Canada). Genotyping was carried out using the Illumina 700 K high density SNP array. Samples of saliva from Italians were collected using buccal swabs rinsed in a 0.9% NaCl solution, using specific purification kits (Invisorb Spin Swab Kit and Genomed; Celbio, Milan, Italy, respectively). Genotyping of the known variant sites (*rs714598*, *rs1726866* and *rs10246939*) was carried out using the KASPar SNP Genotyping Assay (Kbioscience, Heddesdon, UK) and the reads were performed with the ABI PRISM 7900HT instrument (Applied Biosystems).

### Data analysis

Three *TAS2R38* SNPs (*rs714598*, *rs1726866*, *rs10246939*) were analyzed in the HGDP, AGVP, Silk Road and Italian samples. The program PHASE^44^ was used to infer *TAS2R38* haplotypes in the Italian and Silk Road subjects. Individuals from the 1000 Genomes Project Phase 1 were used as a reference to impute *rs714598* in both the Silk Road and HGDP populations and *rs10246939* in some individuals of the AGVP population (N = 1,200), since these SNPs were not present in the genotyping arrays used to type these individuals. Only haplotypes with posterior probability of 0.9 or above were considered. VCF files containing sequence data at the *TAS2R38* locus and its 500 bp upstream and downstream regions (7:141972131-141974273, GRCh38) were retrieved from both the 1000 Genomes Phase 3 Database (ftp://ftp.1000genomes.ebi.ac.uk/vol1/ftp/release/20130502/) and from the 50 and 30-fold coverage Neanderthal and Denisovan genomes aligned to the hg19 human reference sequence, after obtaining the genomic coordinates as appropriate (http://cdna.eva.mpg.de/neandertal/altai/AltaiNeandertal/VCF/ and http://cdna.eva.mpg.de/denisova/VCF/hg19_1000g/) using VCFtools^45^.

The software Arlequin v.3.5^46^ was used to calculate basic population genetics statistics, such as nucleotide diversity, number of polymorphic sites and estimated heterozygosity, and to perform the Analysis of Molecular Variance (AMOVA). Allele frequencies between populations were compared performing Chi-square tests, and the Bonferroni correction was used to correct the nominal P-values (i.e. adjusted P = P value x number of individual tests), with the software PLINK v 1.07^47^. This software was also used to calculate pairwise LD measures and to estimate haplotype blocks which were also confirmed with Haploview^48^. For this analysis, we expanded the analyzed region to 50,000 base pairs in both directions. The haplotype network was constructed with Network 4.5^49^ using a median-joining algorithm.

### Testing natural selection

Departures from neutrality were tested using several approaches: we calculated Tajima’s D values using the DNASP package^50^ in *TAS2R38* and surrounding regions. In addition, we compared these values to the ones calculated across the genome for coding loci with sizes similar to *TAS2R38* (e.g. 1,143 bases) and to those calculated in candidate genes selected from literature known to have undergone some kind of selective pressure. Since it has been shown that Tajima’s test may not be powerful enough to detect departures from neutrality in regions shorter than 5,000 bases^51^, Tajima’s D values were also calculated in a 10,000 bases region encompassing the selected loci. Pairwise F_ST_ values for all pairs of populations and within the same sub-population for *TAS2R38* and surrounding regions were calculated with the software Arlequin v.3.5^46^ and the significance of these statistics was tested using a coalescent simulation adapted from Hudson^52^. In addition, we calculated F_ST_ values for the three *TAS2R38* common SNPs. These values were then compared to the ones calculated across the genome for SNPs of similar frequency (e.g. MAF ranging from 0.42 to 0.47) and to the genome-wide F_ST_ distribution. In order to further explore the signatures of natural selection at *TAS2R38*, we performed Li’s MFDM test^53^, which has been shown to be very robust in distinguishing selection from demography, even in presence of balancing selection. In addition, the Bayesian regression method implemented in Bayescan v2.1^54^ and the HKA test^55^ were applied to our dataset. Finally, simulations of the evolution *TAS2R38* haplotypes outside Africa were performed with the python library simuPOP v1.1.4^56^. We simulated several scenarios for the six *TAS2R38* haplotypes with increasing level of expansions (i.e. 10 fold, 20 fold and 40 fold) and different natural selection pressures (i.e. s = 0.05, s = 0.01 and s = 0.001). Each scenario was replicated 1000 times to generate a prediction of the resulting haplotype frequencies. Finally, sliding-window analyses (100 kb) of heterozygosity were calculated on the entire chromosome 7 in the the 1000 Genome Project populations. More details on the analytical methods can be found in the Supplementary Information.

## Additional Information

**How to cite this article**: Risso, D. S. *et al.* Global diversity in the *TAS2R38* bitter taste receptor: revisiting a classic evolutionary PROPosal. *Sci. Rep.* **6**, 25506; doi: 10.1038/srep25506 (2016).

## Supplementary Material

Supplementary Information

## Figures and Tables

**Table 1:** Detailed distributions of *TAS2R38* haplotypes in the studied populations.

*Population*	PAV	AVI	AAV	AVV	PAI	PVI	AAI	PVV
All	50.76%	42.70%	2.48%	0.32%	0.18%	0.07%	3.39%	0.10%
Africans	50.76%	35.18%	0.61%	0.08%	0.00%	0.15%	13.22%	0.00%
Asians	64.51%	35.31%	0.00%	0.17%	0.00%	0.00%	0.00%	0.00%
Europeans	45.66%	49.22%	3.56%	0.49%	0.32%	0.03%	0.55%	0.17%
Americans	68.61%	26.69%	2.26%	0.00%	0.00%	0.19%	2.26%	0.00%
