# Corrigendum: Global diversity in the *TAS2R38* bitter taste receptor: revisiting a classic evolutionary PROPosal

**DOI:** 10.1038/srep28406

**Published:** 2016-06-27

**Authors:** Davide S. Risso, Massimo Mezzavilla, Luca Pagani, Antonietta Robino, Gabriella Morini, Sergio Tofanelli, Maura Carrai, Daniele Campa, Roberto Barale, Fabio Caradonna, Paolo Gasparini, Donata Luiselli, Stephen Wooding, Dennis Drayna

Scientific Reports 6: Article number: 2550610.1038/srep25506; published online: 05032016; updated: 06272016.

In this Article, all instances of “*rs714598*” should read “*rs713598*”.

