# Supplementary material for: Global diversity in the TAS2R38 bitter taste receptor: revisiting a classic evolutionary PROPosal
Source: Sci Rep. 2016 May 3;6:25506. doi: 10.1038/srep25506 (PMC4853779; doi:10.1038/srep25506)
Supplement: Supplementary Information [file srep25506-s1.pdf]

Supplementary Information

Global diversity in the *TAS2R38* bitter taste receptor: revisiting a classic evolutionary PROPosal

Davide S. Risso, Massimo Mezzavilla, Luca Pagani, Antonietta Robino, Gabriella Morini, Sergio Tofanelli,

Maura Carrai, Daniele Campa, Roberto Barale, Fabio Caradonna, Paolo Gasparini, Donata Luiselli,

Stephen Wooding and Dennis Drayna

Supplementary Figure S1. F_{ST} calculated for all SNPs across the genome having MAF similar to ones of *TAS2R38* variants (e.g. MAF ranging from 0.42 to 0.47) in the 1000 Genomes dataset. Arrows indicate the position of *TAS2R38* SNPs *rs10246939*, *rs714598* and *rs1726866* respectively in global (A) and African (B) populations.

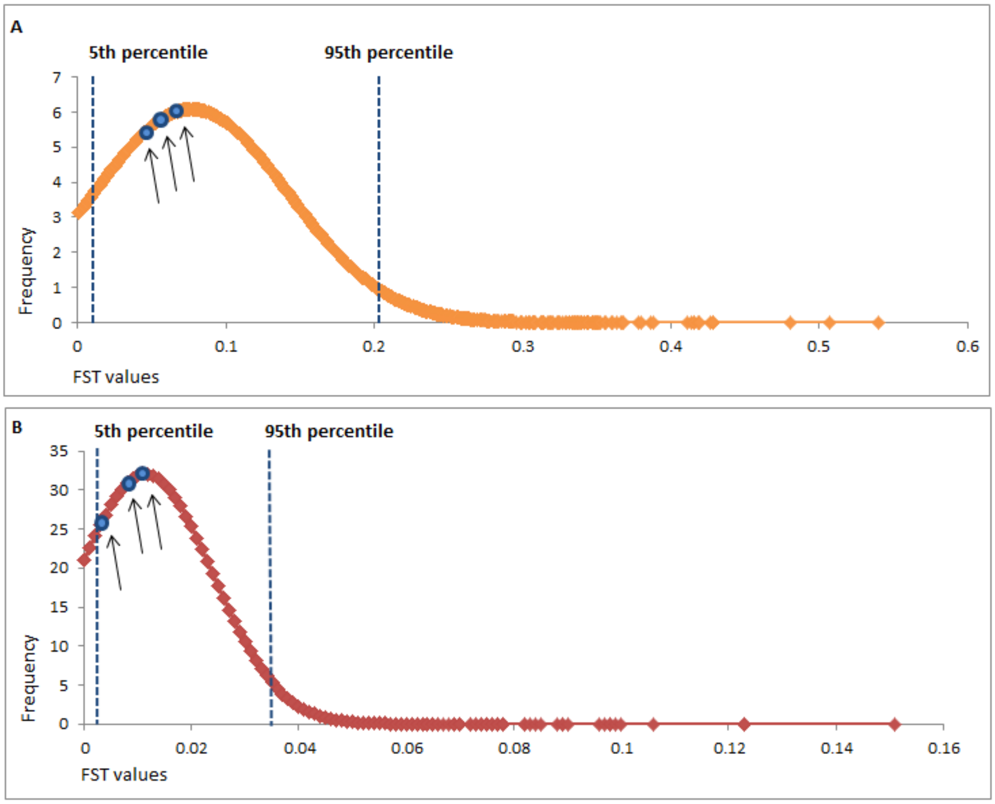

Supplementary Figure S2. Details of frequency distributions of TAS2R38 AAI diplotypes in the studied African populations.This map has been modified from its original version (https://commons.wikimedia.org/wiki/File:BlankMap-Africa.svg).

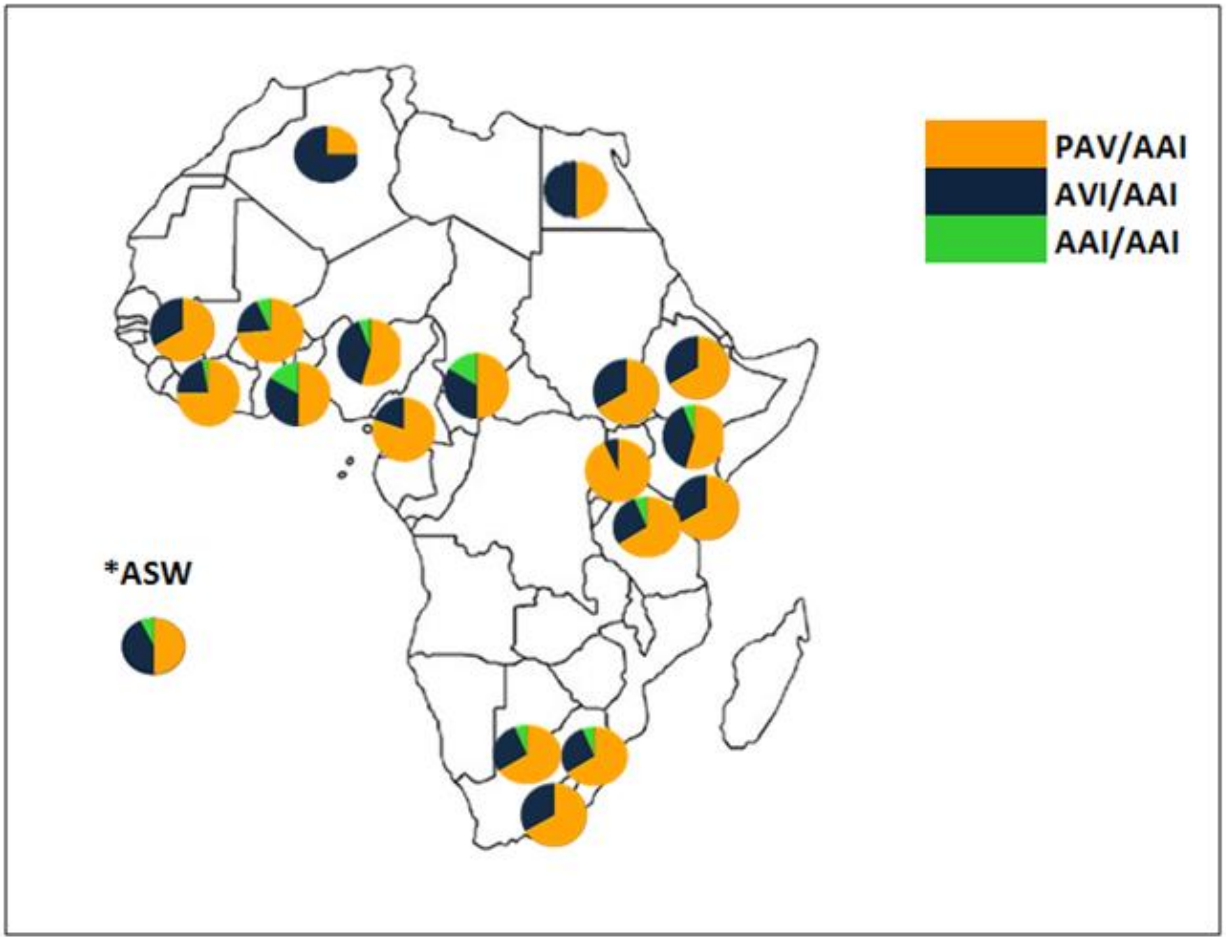

Supplementary Figure S3. Tajima's D values calculated for all loci across the genome having similar size to *TAS2R38* (e.g. 1,143 bases) in the 1000 Genomes dataset. Arrows indicate the position of *TAS2R38* in non-Africans (A) and African (B) populations.

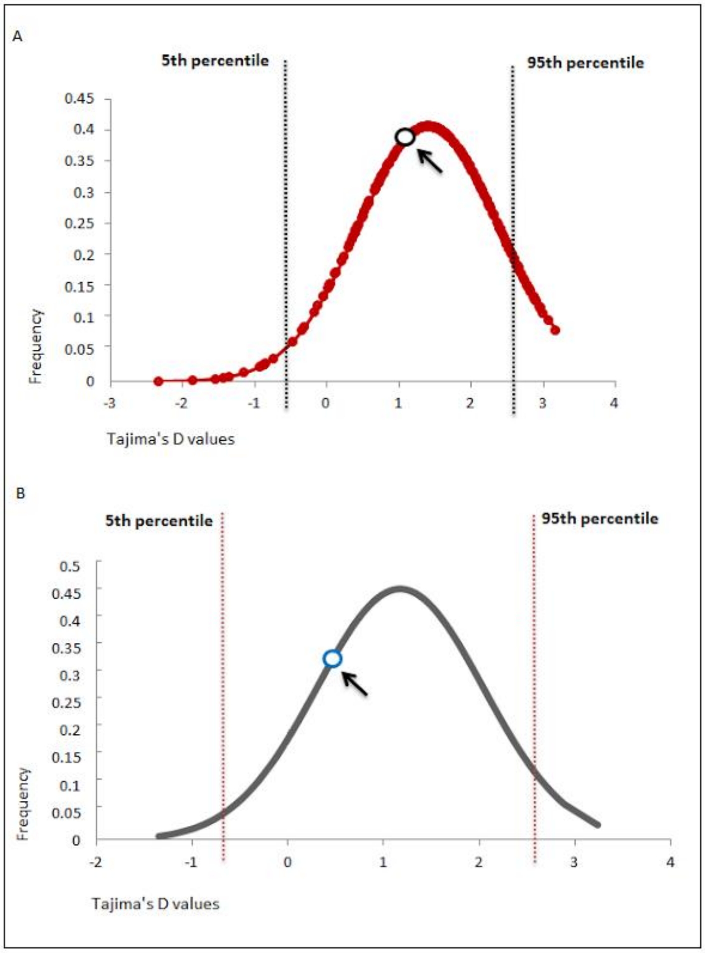

Supplementary Figure S4. Simulated *TAS2R38* haplotype frequencies under balancing selection (s=0.001) acting on PAV/AVI before the Out Of Africa event in African (A), Europeans (B), Asian (C) and American (D) populations.

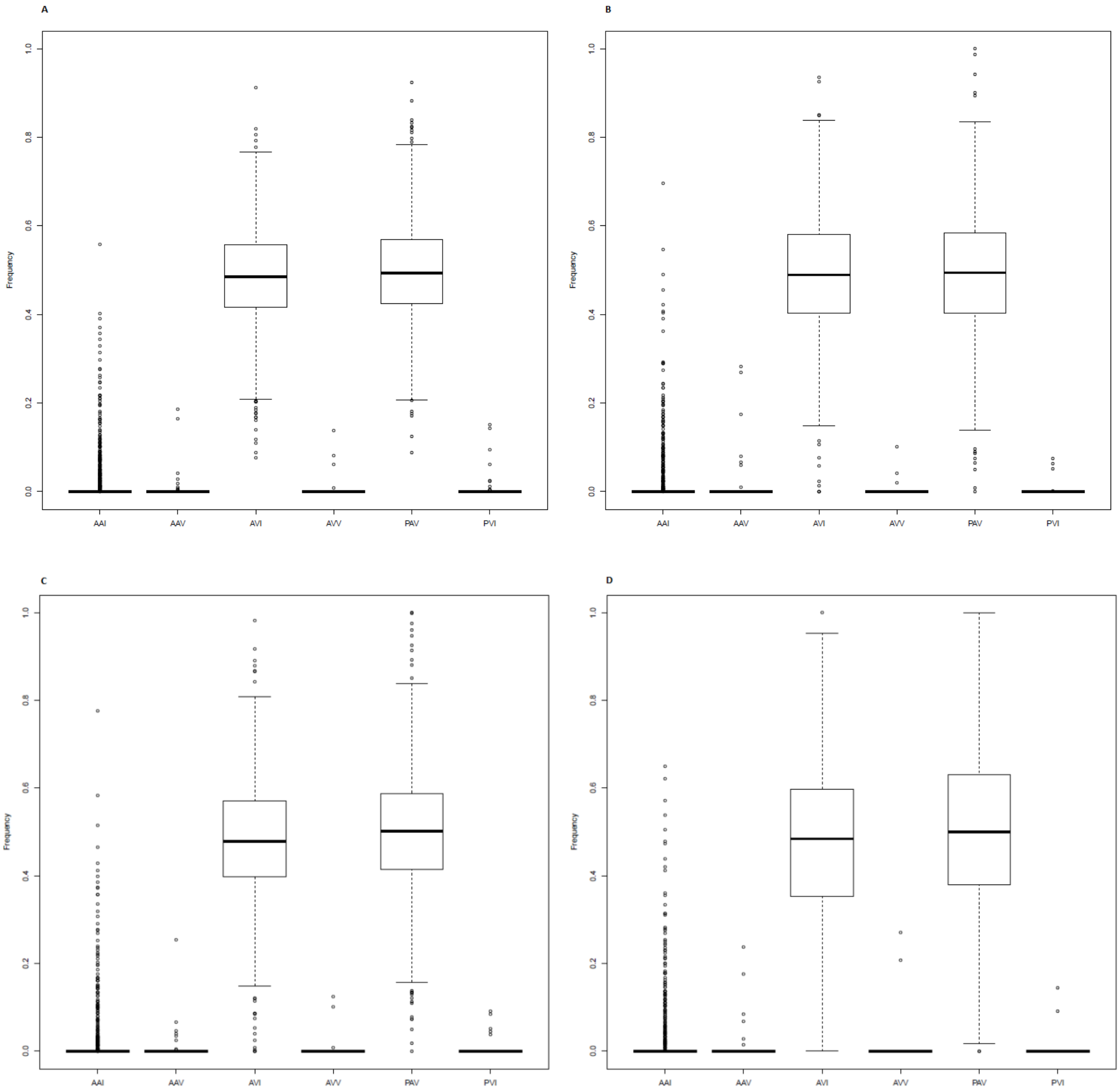

Supplementary Figure S5. Simulated *TAS2R38* haplotype frequencies under balancing selection (s=0.001) on PAV/AVI and directional selection (s=0.0001) acting in African individuals before the Out Of Africa event.

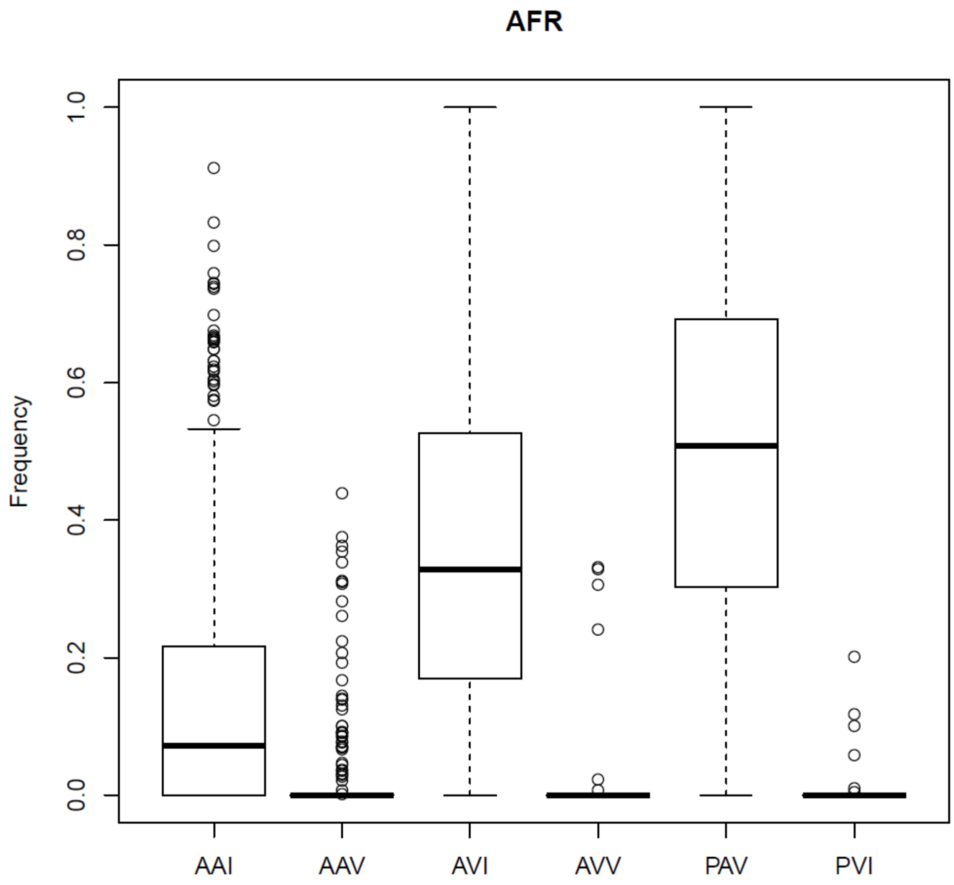

Supplementary Table S1. Polymorphic sites, gene and nucleotide diversity at the *TAS2R38* gene in the analyzed African, Asian, European and Latin American populations.

ASW, Americans of African Ancestry in SW USA; LWK, Luhya in Webuye, Kenya; YRI, Yoruba in Ibadan, Nigeria; MSL, Mende in Sierra Leone; ESN, Esan in Nigeria; CHB, Han Chinese in Bejing, China; CHS, Southern Han Chinese; JPT, Japanese in Tokyo, Japan; CEU, Utah Residents (CEPH) with Northern and Western European Ancestry; FIN, Finnish in Finland; GBR, British in England and Scotland; IBS, Iberian Population in Spain; TSI, Toscani in Italia; CLM, Colombians from Medellin, Colombia; MXL, Mexican Ancestry from Los Angeles USA; PUR, Puerto Ricans from Puerto Rico; PEL, Peruvians from Lima, Peru.

	**ASW**	**LWK**	**YRI**	**MSL**	**ESN**
polymorphic sites	11	13	14	17	14
gene diversity	0.75 +/- 0.02	0.81 +/- 0.01	0.79 +/- 0.01	0.78 +/- 0.02	0.79 +/- 0.01
nucleotide diversity	0.07 +/- 0.03	0.07 +/- 0.03	0.07 +/- 0.04	0.07 +/- 0.03	0.07 +/- 0.03
	**CHB**	**CHS**	**JPT**		
polymorphic sites	5	5	5		
gene diversity	0.48 +/- 0.02	0.47 +/- 0.02	0.55 +/- 0.01		
nucleotide diversity	0.05 +/- 0.03	0.05 +/- 0.03	0.05+/- 0.04		
	**CEU**	**FIN**	**GBR**	**IBS**	**TSI**
polymorphic sites	4	7	4	4	5
gene diversity	0.55 +/- 0.01	0.53 +/- 0.02	0.50 +/- 0.02	0.51+/- 0.06	0.55 +/- 0.01
nucleotide diversity	0.05 +/- 0.04	0.05 +/- 0.03	0.05 +/- 0.04	0.05 +/- 0.04	0.05 +/- 0.03
	**CLM**	**MXL**	**PUR**	**PEL**	
polymorphic sites	6	9	8	9	
gene diversity	0.57 +/- 0.02	0.56 +/- 0.03	0.62+/- 0.03	0.45 +/- 0.04	
nucleotide diversity	0.05 +/- 0.03	0.05 +/- 0.03	0.05 +/- 0.03	0.05 +/- 0.03	

Supplementary Table S2. Fifth, 25th, 75th, 95th quartiles and average and maximum heterozygosity values calculated with sliding-window analyses (100kb) on chromosome 7 in the analyzed 1000 Genomes populations. TAS2R38 indicates values for the window containing *TAS2R38* gene.

ASW, Americans of African Ancestry in SW USA; CEU, Utah Residents (CEPH) with Northern and Western European Ancestry; CHB, Han Chinese in Bejing, China; CHS, Southern Han Chinese; CLM, Colombians from Medellin, Colombia; ESN, Esan in Nigeria; FIN, Finnish in Finland; GBR, British in England and Scotland; IBS, Iberian Population in Spain; JPT, Japanese in Tokyo, Japan; LWK, Luhya in Webuye, Kenya; MSL, Mende in Sierra Leone; MXL, Mexican Ancestry from Los Angeles USA; PEL, Peruvians from Lima, Peru; PUR, Puerto Ricans from Puerto Rico; TSI, Toscani in Italia; YRI, Yoruba in Ibadan, Nigeria.

**POP**	**5%**	**25%**	**Average**	**75%**	**95%**	**Max**	***TAS2R38***
ASW	0.16	0.19	0.21	0.24	0.27	0.33	0.23
CEU	0.09	0.15	0.18	0.20	0.24	0.31	0.18
CHB	0.07	0.13	0.17	0.19	0.23	0.32	0.20
CHS	0.07	0.13	0.17	0.19	0.24	0.32	0.20
CLM	0.11	0.16	0.19	0.21	0.25	0.33	0.23
ESN	0.16	0.19	0.21	0.23	0.27	0.35	0.22
FIN	0.09	0.15	0.18	0.20	0.24	0.32	0.20
GBR	0.09	0.15	0.18	0.20	0.24	0.32	0.19
IBS	0.09	0.15	0.18	0.20	0.25	0.31	0.20
JPT	0.07	0.13	0.16	0.19	0.24	0.32	0.18
LWK	0.15	0.19	0.21	0.23	0.27	0.35	0.23
MSL	0.16	0.19	0.21	0.24	0.27	0.34	0.23
MXL	0.10	0.15	0.18	0.21	0.24	0.31	0.23
PEL	0.08	0.13	0.16	0.19	0.24	0.32	0.22
PUR	0.12	0.16	0.19	0.21	0.25	0.32	0.22
TSI	0.09	0.15	0.18	0.20	0.24	0.31	0.20
YRI	0.16	0.19	0.21	0.23	0.27	0.35	0.23

Supplementary Table S3. Complete list of the analyzed populations.

Continent	Population ID	Country	N Chromosomes	Continent	Population ID	Country	N Chromosomes
Africa	ASW	African Ancestry	122	Asia	Khorog	Tajikistan	32
Africa	Bantu Kenya	Kenya	22	Asia	Lahu	China	16
Africa	Bantu South Africa	Angola	16	Asia	Makrani	Pakistan	50
Africa	Biaka Pygmy	Central African Republic	42	Asia	Martumi	Armenia	30
Africa	ESN	Nigeria	198	Asia	Miao	China	20
Africa	LWK	Kenya	194	Asia	Mongola	China	20
Africa	Mandenka	Senegal	220	Asia	Mtskheta Mtianeti	Georgia	32
Africa	MbutiPygmy	Congo	26	Asia	Naxi	China	16
Africa	Mozabite	Algeria-Mzab	58	Asia	Oroqen	China	18
Africa	MSL	Sierra Leone	170	Asia	Palestinian	Israel-Central	92
Africa	San	Namibia	12	Asia	Pathan	Pakistan	44
Africa	Yoruba	Nigeria	220	Asia	Rushan	Tajikistan	26
Africa	Zeravshan	Tajikistan	18	Asia	She	China	20
Africa	Ethiopia	Ethiopia	256	Asia	Shing	Tajikistan	30
Africa	Egypt	Egypt	200	Asia	Sindhi	Pakistan	48
Africa	Baganda	Uganda	200	Asia	Sis	Azerbaijan	44
Africa	Banyarwanda	Uganda	200	Asia	Tashkent	Uzbekistan	34
Africa	Barundi	Burundi	194	Asia	Tu	China	20
Africa	Fula	Senegal	148	Asia	Tujia	China	20
Africa	Ga-Adangbe	Ghana	200	Asia	Uygur	China	20
Africa	Igbo	Nigeria	198	Asia	Xibo	China	18
Africa	Jola	Senegal	158	Asia	Yakut	Siberia	50
Africa	Kalenjin	Kenya	200	Asia	Yegvard	Armenia	28
Africa	Kikuyu	Kenya	198	Asia	Yerevan	Armenia	18
Africa	Sotho	South Africa	172	Asia	Yi	China	20
Africa	Wolof	South Africa	156	Europe	Adygei	Russia-Caucasus	34
Africa	Zulu	South Africa	200	Europe	Basque	France	48
Asia	Alga	Kazakhstan	60	Europe	CEU	NorthWest Europe	170
Asia	Almaty	Kazakhstan	62	Europe	FIN	Finland	186
Asia	Balochi	Pakistan	48	Europe	French	France	56
Asia	Bedouin	Israel-Negev	92	Europe	GBR	Great Britain	178
Asia	Brahui	Pakistan	50	Europe	IBS	Spain	28
Asia	Bukhara	Uzbekistan	70	Europe	Orcadian	Orkney Islands	30
Asia	Burusho	Pakistan	50	Europe	Russian	Russia	50
Asia	Cambodian	Cambodia	20	Europe	Sardinians	Italy	56
Asia	Chambarak	Armenia	56	Europe	Tuscany	Italy	1408
Asia	CHS	China	200	Europe	N-Italians	Italy	24
Asia	Dai	China	20	Europe	Calabria	Italy	142
Asia	Daur	China	18	Europe	Emilia-Romagna	Italy	128
Asia	Deprabak	Armenia	40	Europe	Lazio	Italy	278
Asia	Druze	Israel-Carmel	84	Europe	Lombardy	Italy	90
Asia	Gavar	Armenia	22	Europe	Sicily	Italy	794
Asia	Han	China	282	Europe	Umbria	Italy	94
Asia	Hazara	Pakistan	44	Europe	Abruzzo	Italy	156
Asia	Hezhen	China	16	North America	Maya	Mexico	42
Asia	Imereti	Georgia	80	North America	Puerto Ricans	Puerto Rico	110
Asia	Ismailly	Azerbaijan	38	Oceania	Melanesian	Melanesia	20
Asia	Japanese	Japan	234	Oceania	Papuan	Papua New Guinea	34
Asia	Kakheti	Georgia	64	South America	Colombians	Colombia	134
Asia	Kalaikhum	Tajikistan	26	South America	Karitiana	Brazil	28
Asia	Kalash	Pakistan	46	South America	Mexicans	Mexico	160
Asia	Karshi	Uzbekistan	28	South America	PEL	Peru	170
				South America	Surui	Brazil	16

Supplementary Information

Using Bayescan to detect departures from neutral expectations

A specific Bayesian approach was used to detect evidence of balancing selection and positive selection or natural selection. For a specific locus, departure from neutrality were inferred using a Bayesian regression method implemented in Bayescan v2.1 (1), Bayescan models the posterior probability for each locus to be under positive or balancing selection. The significance of the selection model was set using a threshold of false discovery rate (FDR) at 0.0001 and then calculated the q-value for each locus. We selected this stringent threshold to minimize false positive results. 20 pilot runs of 5000 iterations were used for the Markov chain Monte Carlo (MCMC) algorithm. A burn-in of 50,000 iterations was used and checked for convergence, followed by 50,000 iterations.

Simulations of haplotype evolution

We simulated several scenarios of *TAS2R38* haplotypes evolution. The initial population effective size (N_e) was set at N=10,000 at 100kya. We then introduced a bottleneck event at 60kya reducing the initial N_e to N=3000. After this event, we introduced, starting at 20kya, an increase of population size with different growth rates (i.e. 10, 20 and 40-fold). We set the time of split between American and East Asian populations at 25kya. These simulated parameters were chosen accordingly to previous studies involving simulations of population demography (2,3,4,5) and all simulations were replicated 1000 times. We set the haplotype distributions observed in present-day African populations as starting frequencies, as follows: AAI (0.1322), AAV (0.0061), AVI (0.3518), AVV (0.0008), PAV (0.5076), PVI (0.0015). The first simulated scenario involved no natural selection.

We subsequently simulated models with different selection coefficient acting on PAV/AVI haplotypes, in order to test the balancing selection hypothesis. Selective pressures were set as s=0.05, s=0.01 and s=0.001, acting both before and after the Out of Africa (OOA) event.

We then further explored the distribution of the AAI haplotype in African populations, with different selective pressures (s=0.05, s=0.01, s=0.001 and s=0.0001) both before and after OOA.

Testing natural selection

We have carefully selected 5 loci from literature (*LCT*, *FOXP2*, *TRPV6*, *EDAR* and *KEL*) known to have been under positive selection (6,7,8,9,10), 5 (*ABO*, *HLA-A*, *HLA-B*, *ERAP2*, *IL10RB*) under balancing selection (10,11,12,13) and 5 regions (1pMB4, XqMB141, 22q11, *VTN*, *LTA*) considered evolutionary neutral (14,15,16). We calculated Tajima's D values for these loci in the 1000 Genomes dataset, in both African and non-African populations, and compared them for the ones calculated for *TAS2R38*. Moreover, we compared these values to the ones calculated across the genome for loci with similar size to *TAS2R38* (e.g. 1,143 bases). In addition, we calculated the F_{ST} statistics for all SNPs across the genome having MAF similar to ones of *TAS2R38* variants (e.g. MAF ranging from 0.42 to 0.47) in the 1000 Genomes dataset. We used the online database SNP@Evolution (17) to calculate the genome-wide estimates of F_{ST} values. A normal, genome-wide, distribution of FST and Tajima's D values was then created and both the 5th and 95th percentiles were calculated.

Supplementary References

1. Foll M, Gaggiotti O. A genome-scan method to identify selected loci appropriate for both dominant and codominant markers: a Bayesian perspective. Genetics. Oct;180(2):977-93 (2008)

2. Gutenkunst, R. N., Hernandez, R. D., Williamson, S. H. & Bustamante, C. D. Inferring the joint demographic history of multiple populations from multidimensional SNP frequency data. PLoS Genet 5, e1000695 (2009)

3. Li, H., Durbin, R. Inference of human population history from individual whole-genome sequences. Nature. Jul 13;475(7357):493-6 (2011)

4. Campbell, MC. et al. Evolution of functionally diverse alleles associated with PTC bitter taste sensitivity in Africa. Mol Biol Evol. 29(4):1141-53 (2012)

5. Mezzavilla, M., Geppert, M., Tyler-Smith, C., Roewer, L. & Xue, Y. Insights into the origin of rare haplogroup C3* Y chromosomes in South America from high-density autosomal SNP genotyping. Forensic Science International: Genetics 15, 115-120 (2015)

6. Bersaglieri, T. et al. Genetic signatures of strong recent positive selection at the lactase gene. Am J Hum Genet. Jun;74(6):1111-20 (2004)

7. Ayub, Q. et al. FOXP2 targets show evidence of positive selection in European populations. Am J Hum Genet. May 2;92(5):696-706 (2013)

8. Hughes, DA. et al. Parallel selection on TRPV6 in human populations. PLoS One. Feb 27;3(2):e1686 (2008)

9. Bryk, J. et al. Positive selection in East Asians for an EDAR allele that enhances NF-kappaB activation. PLoS One. May 21;3(5):e2209 (2008)

10. Akey, JM. Et al. Population history and natural selection shape patterns of genetic variation in 132 genes. PLoS Biol. Oct;2(10):e286 (2004)

11. Saitou N, Yamamoto F. Evolution of primate ABO blood group genes and their homologous genes. Mol Biol Evol. Apr;14(4):399-411 (1997)

12. Hedrick, PW., Thomson, G. Evidence for balancing selection at HLA. Genetics. Jul;104(3):449-56 (1983)

13. Andrés, AM., et al. Balancing selection maintains a form of ERAP2 that undergoes nonsense-mediated decay and affects antigen presentation. PLoS Genet. Oct 14;6(10):e1001157 (2010)

14. Wall, JD., et al. A novel DNA sequence database for analyzing human demographic history. Genome Res. Aug;18(8):1354-61 (2008)

15. Zhao, Z., et al. Worldwide DNA sequence variation in a 10-kilobase noncoding region on human chromosome Proc Natl Acad Sci USA. Oct 10;97(21):11354-8 (2000)

16. Wang, X., Thomas, SD., Zhang, J. Relaxation of selective constraint and loss of function in the evolution of human bitter taste receptor genes. Hum Mol Genet. Nov 1;13(21):2671-8 (2004)

17. Cheng, F., Chen, W., Richards, E., Deng, L., Zeng, C. SNP@Evolution: a hierarchical database of positive selection on the human genome. BMC Evol Biol. 9:221 (2009)
